# Long-term outcomes of neuromuscular retraining after hypoglossal to facial nerve transfer for facial palsy

**DOI:** 10.1007/s00405-026-10247-3

**Published:** 2026-04-23

**Authors:** Ignacio Javier Fernandez, Alice Barbazza, Edoardo Serafini, Claudio Melchiorri, Monica Guidotti, Federica Nizzoli, Marco Bonali

**Affiliations:** 1 Department of Medical and Surgical Sciences (DIMEC), University of Bologna, Bologna, Italy; 2 Otolaryngology Head and Neck Surgery Department of Ravenna, Ravenna Hospital, AUSL Romagna, Ravenna, Italy; 3 IRCCS Azienda Ospedaliero - Universitaria Di Bologna (Otolaryngology and Audiology Department of the University Hospital of Bologna), Bologna, Italy; 4 Otolaryngology Head and Neck Surgery Department of Rimini, Rimini Hospital, AUSL Romagna, Rimini, Italy; 5 Otolaryngology Head and Neck Surgery Department, University Hospital of Modena, Modena University, Modena, Italy

**Keywords:** Facial paralysis, Hypoglossal to facial nerve coaptation, Neuromuscular retraining

## Abstract

**Purpose:**

This retrospective multicenter cohort study aims to evaluate the long-term outcomes of neuromuscular retraining (NMR) following primary end-to-end hypoglossal-facial nerve coaptation (HFC) compared with alternative rehabilitation methods, conducted across two tertiary referral centers in Italy.

**Methods:**

Patients undergoing end-to-end HFC as the sole surgical procedure for facial reanimation between 2009 and 2023 at two university hospitals were included. Patients receiving NMR were compared with controls rehabilitated through other techniques. Facial function was assessed using the House-Brackmann (HB) and Sunnybrook scales at standardized intervals during the rehabilitation period up to 24 months postoperatively. Patients had a minimum clinical follow-up of 18 months. Patient-reported outcomes were evaluated via the Facial Disability Index (FDI) and MD Anderson Dysphagia Inventory (MDADI).

**Results:**

Thirty-nine patients were included (30 NMR group, 9 control group). NMR patients showed significant improvements in all Sunnybrook scale domains compared with controls (p < 0.001), particularly in symmetry at rest and synkinesis reduction. Improvement in voluntary movement continued up to 18 months. Sex-specific analysis indicated better synkinesis control in females beyond 12 months, although not statistically significant. Overall FDI and MDADI scores were very good, but not significantly different between groups. No significant influence of age, or time to surgery on outcomes was observed.

**Conclusion:**

Specifically designed long-term NMR following end-to-end HFC significantly enhances facial reanimation outcomes, promoting cortical plasticity and limiting synkinesis. Long-term structured rehabilitation should be considered essential for optimal recovery after HFC.

## Introduction

Hypoglossal-to-facial nerve coaptation (HFC), first proposed by Körte in 1903 [1, 2], is a reanimation procedure designed to restore facial muscle movement in cases of facial paralysis. Variations of the surgical technique, such as end-to-side HFC, "jump" HFC, and the split technique, have been described [3–6]. Several factors contribute to the effectiveness of these procedures. Firstly, the motor cortex areas for the XII and VII cranial nerves are large and contiguous [7]. Secondly, both nerves possess a motor reflex arc involving sensory stimuli from the V cranial nerve. Thirdly, both nerves maintain resting tone activity. Lastly, they act synergistically in coordinating mimetic and prandial functions and contain myelinated motor fibers with similar fascicular anatomy. The popularity of the end-to-end HFC has been partially limited due to postoperative tongue morbidity [8, 9]. Rehabilitation programs including specific lingual exercises may help patients adapt to the new motor circuitry and reduce functional impairment [10]. Among the various rehabilitation strategies, Neuromuscular Retraining (NMR) has demonstrated the ability to reduce synkinesis after facial palsy and improve facial motor control, leading to better functional outcomes [11–13].

The aim of this study is to present our experience with primary end-to-end HFC followed by rehabilitation based on NMR, with long-term follow-up, and to compare the results with a control group not treated with NMR after surgery.

## Methods

### Patient selection

All patients undergoing end-to-end hypoglossal-to-facial nerve transfer (HFC) at two tertiary referral centers in Italy between February 2009 and November 2023 were included in the study population. Inclusion criteria were age > 18 years, acceptance to undergo NMR treatment program and end-to-end HFC as the sole surgical procedure for facial reanimation within 18 months from facial palsy onset. Demographic data and data related to facial nerve paralysis were collected from patients’ medical records. Patients were excluded if they were lost before a minimum postoperative follow-up of 18 months after the HFC procedure, or if they showed no adherence to the postoperative rehabilitation program. Functional outcomes were longitudinally assessed using the House–Brackmann and Sunnybrook scales up to 24 months postoperatively, while patient-reported outcome measures (FDI and MDADI) were administered at the 24-month follow-up visit. Patients undergoing botulinum toxin injection or other surgical procedures after HFC were excluded from the study. Those who underwent rehabilitation in our institutions were treated with NMR by two experienced speech therapists. This retrospective multicenter study was approved by the local Institutional Review Boards (IRB) of the participating centers (approval numbers 901/2021 and 160/2022). Indications for end-to-end HFC were severe facial nerve paralysis (House–Brackmann grade V–VI) in patients with an anatomically preserved extrapetrosal facial nerve and no improvement after rehabilitation within 18 months from paralysis onset. In selected cases involving tumors affecting the facial nerve (such as geniculate ganglion hemangioma, facial nerve schwannoma, or endolymphatic sac tumor), immediate HFC reconstruction was performed during the same surgical procedure, even in patients presenting with House–Brackmann grade IV paralysis. Patients in the study group underwent scheduled assessment of the facial palsy with both House-Brackmann and Sunnybrook scales before surgery, after surgery (T0), 6 months (T1), 12 months (T2), 18 months (T3) and 24 months (T4).

### Control group

The control group consisted of patients who underwent hypoglossal–facial nerve transfer at our institutions but completed postoperative rehabilitation outside our centers. These patients were treated with heterogeneous rehabilitation approaches, including active exercises, electrostimulation, Kabat therapy, or combinations thereof. Detailed information regarding the number of sessions, intensity, therapist qualifications, and patient adherence was not systematically available.

This group therefore represents a pragmatic real-world comparator reflecting non-standardized rehabilitation practices. Owing to its heterogeneity, the control group does not permit direct attribution of outcome differences to specific rehabilitation methods, and comparisons with the NMR group should be interpreted as exploratory.

These patients were recalled to our facial nerve outpatient clinic and underwent assessment both with HB and Sunnybrook scales. They were finally included if they had undergone the surgical procedure at least 24 months before and had completed their rehabilitation therapy.

Patients of the study and control group were asked to complete the MDADI (MD Anderson dysphagia inventory) and FDI (facial disability index) questionnaires after 24 months of rehabilitation [14, 15].

### Surgical procedure

All procedures were performed by two senior surgeons with extensive experience in hypoglossal-to-facial nerve transfer. Facial nerve dissection was performed proximally to secure 2–3 cm of the facial nerve, which is cut in front of the stylomastoid foramen and turned over for coaptation with the hypoglossal nerve. The hypoglossal nerve was identified and dissected along the posterior belly of the digastric muscle. The hypoglossal descending branch was identified and interrupted. The main trunk of the hypoglossal nerve was dissected and cut distally, just before it entered the mylohyoid muscle. It was then rotated superiorly, and the proximal stump of the hypoglossal nerve was coaptated with the distal stump of the facial nerve through epineural suture with 9–0 nonabsorbable monofilament. Fibrin glue is then applied to secure the micro-suture, which was eventually covered with a fragment of vein.

### Postoperative management

Tongue exercises were initiated the day after the surgical procedure and practiced daily. Initially, the required movements were rough, but after four to six months, when hypoglossal axon regrowth reached the facial muscles, patients began performing the movements with increasing precision. Follow-up visits were performed monthly for the first year and every two months thereafter until 36 months. Facial paralysis grades were recorded according to the H-B and Sunnybrook scales at fixed timepoints (T0, T1, T2, T3, T4).

### NMR adapatation for hypoglossal facial nerve transfer

NMR, developed in 1997 by Diels and Combs [11], was adapted to patients undergoing hypoglossal–facial nerve coaptation and delivered by certified speech therapists (logopedists). It is based on external and intraoral massages performed in front of a mirror to allow patients to develop cognitive control of facial movements. Slow movements allow patients to observe the exercise, to modify its speed and strength, thereby improving motor control and inhibiting synkinesis. Small movements promote the contraction of target muscles, limit the hyperactivity of adjacent muscles and improve overall muscle coordination. Symmetrical movements reinforce physiological responses and prevent hypercontraction of the healthy side. Exercises are carried out initially passively, then passively-active and finally actively, covering all facial areas with particular attention to eye closure and the movement of the levator labii superioris and zygomaticus muscles (smile) [11–13].

Sessions were conducted once per month, each lasting approximately 30–45 min, for a maximum duration of 24 months. Rehabilitation combined supervised in-clinic sessions with structured home-based exercises. Home exercises were prescribed daily. Lingual exercises were performed twice per exercise, 3–4 times per day, while facial exercises were performed twice per exercise, 5 times per day. Adherence was considered high based on therapist reports during follow-up visits.

Rehabilitation progressed through three stages: (1) active lingual activation to elicit facial movement, (2) progressive reduction of tongue movement with cognitive initiation of facial contraction, and (3) cortical dissociation, in which facial movements were generated by direct voluntary intent without tongue activation.

### Statistical analysis

The statistical analysis of the results was performed using 24.0 SPSS for Windows (IBM SPSS Statistics, Chicago, USA). Continuous variables were expressed as mean ± standard deviation (SD). The repeated measures ANOVA analysis was used for comparison of continuous variables at different timepoints among patients. All the ANOVA analyses were run using Sunnybrook and House Brackmann measures in the timepoints (T0, T1, T2, T3, T4) during rehabilitation as the repeated measures factors, with age and time between paralysis and surgery as covariates. For within subject effects, Maunchly’s test of sphericity was used to assess sphericity violation and the Greenhouse–Geisser sphericity correction was used when necessary. Sex was included as an additional between-subject factor. Comparisons between the study and control groups, were conducted using Student’s t-test for continuous variables with a normal distribution and Mann–Whitney U Test for non-normally distributed continuous variables. Comparisons between groups were performed by Pearson’s chi-square or Fischer exact test for discrete variables, as appropriate. The results were regarded as statistically significant for p values < 0.05 with a confidence interval of 95%.

## Results

Thirty-nine patients were included in the study. The study group included 30 patients (76,9%) rehabilitated NMR and the control group consisted of 9 patients (23,97%) rehabilitated with other techniques. The most common aetiology of the paralysis was iatrogenic injury after vestibular schwannoma surgery (58,9%). More than half of cases (66,6%) presented with severe facial palsy, 8 patients (20,5%) presented with grade V and 5 patients (12,8%) presented with grade IV. The latter were patients affected by geniculate ganglion haemangioma (3 cases), endolymphatic sac tumour (1 case) and facial nerve Schwannoma (1 case), in which the HFC were performed during the same operation for the tumour removal. The perioperative complications were 1 dehiscence of the closure of the external auditory canal (not directly related with the HFC procedure), 2 cases of post-operative bleeding, and 1 case of wound infection. As regards the VII nerve rehabilitation and the rehabilitation of the tongue, study group and control group data are reported in Table 1.

**Table 1:** Population data

	**Patients with NMR rehabilitation (Study group)**	**Patients without NMR rehabilitation (Control group)**
**Age**		
Mean [Min—Max]	51.967 (13.707) [26—77]	43.222 (16.084) [16—62]
**Side**	**N (%)**	**N (%)**
Right	17 (56)	3 (33)
Left	13 (43)	6 (66)
**Sex**	**N (%)**	**N (%)**
M	16 (53)	5 (55)
F	14 (46)	4 (44)
Missing	0 (0)	0 (0)
Total	30 (100)	9 (100)
**Paralysis Etiology**	**N (%)**	**N (%)**
Schwannoma VIII c.n	17 (56)	6 (66)
Schwannoma VII c.n	3 (10)	2 (22)
Haemangioma	4 (13)	0 (0)
Meningioma	2 (6)	1 (11)
Cholesteatoma petrous apex	2 (6)	0 (0)
Papillary endolymphatic sac tumour	1 (3)	0 (0)
Herpes Zoster Virus	1 (3)	0 (0)
Missing	0 (0)	0 (0)
Total	30 (100)	9 (100)
**Time between paralysis and anastomosis (days)**		
Mean (DS) [Min—Max]	238.633 (192.045) [25.000–730.000]	122.556 (118.303) [0.000–374.000]
**Type of facial rehabilitation**	**N (%)**	**N (%)**
NMR	30 (100)	0 (0)
Electrostimulation	0 (0)	1 (11)
Electrostimulation and active rehabilitation	0 (0)	5 (55)
Missing	0 (0)	3 (33)
Total	30 (100)	9 (100)
**Type of hypoglossal rehabilitation**	**N (%)**	**N (%)**
Speech therapist	30 (100)	1 (11)
Other	0 (0)	6 (66)
Missing	0 (0)	2 (22)
Total	30 (100)	9 (100)

Abbreviations: N: number of patients; %: percentage of patients

### Evolution of facial palsy over time in the study group

**Fig. 1:**
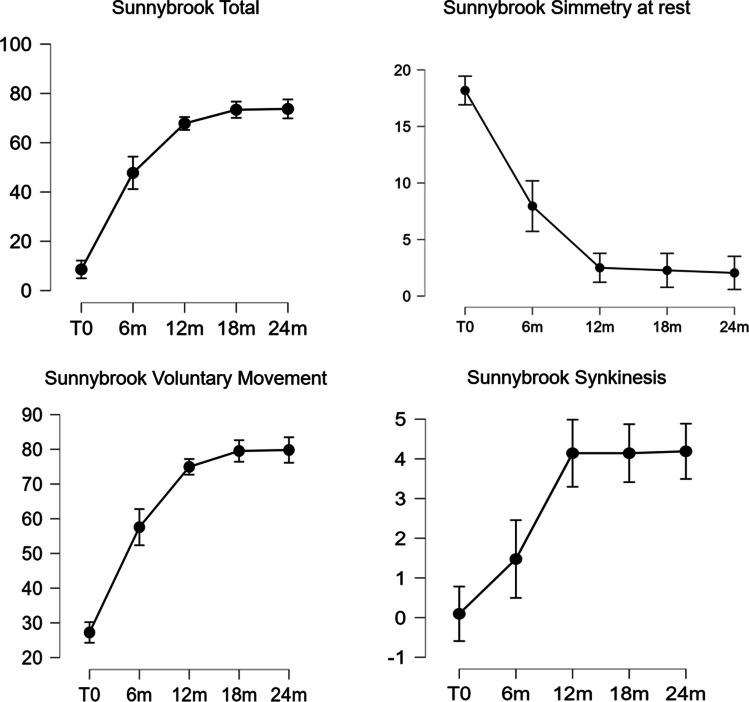
Evolution of facial palsy over time in the study group. **T0**: sunnybrook score before surgery. **6 m, 12 m, 18 m, 24 m**: sunnybrook score after 6, 12, 18 and 24 months of rehabilitation with NMR

**Fig. 2:**
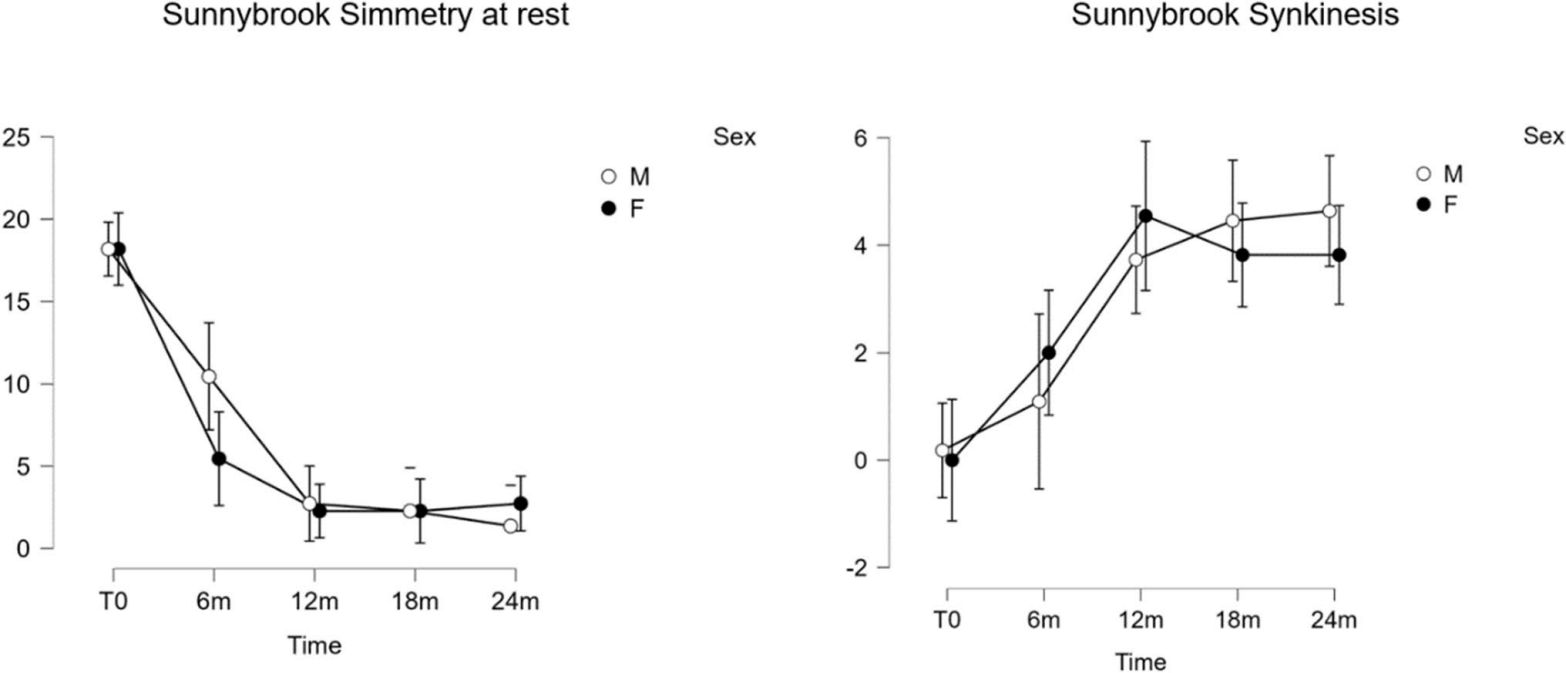
Evolution of symmetry at rest and synkinesis among genders. **T0**: before surgery. **6 m, 12 m, 18 m, 24 m**: Sunnybrook score after 6, 12, 18 and 24 months of rehabilitation with NMR. **M**: Male; **F**: Female

The mean follow-up time was 22 months (range 18–36 months). ANOVA modelling revealed significant improvements in facial palsy severity during rehabilitation as shown in Table 2. In the early stages of recovery (0–12 months) almost all recovery of symmetry at rest was observed, achieving maximum values at 12 months and remaining stable thereafter. Voluntary movement showed main improvements within the first 12 months, followed by a mild improvement from 12 to 18 months and a stable trend from 18 to 24 months. Synkinesis scores showed no statistically significant time-related change across the rehabilitation period. An initial worsening between 6 and 12 months was observed, consistent with the known phase of aberrant reinnervation, followed by stabilization between 12 and 24 months. These findings indicate that NMR did not significantly reduce synkinesis over time but appeared to limit its progression after the first year (Fig. 1). Sex-corrected data for Sunnybrook symmetry at rest showed a more rapid recovery in female patients within the first 12 months, while males displayed a more gradual trend. A similar pattern was found in the synkinesis score: females improved more significantly, especially between 12 and 24 months (Fig. 2).

**Table 2:** Repeated measures ANOVA (Study group)

Sunnybrook	Within Subjects					Between Subjects				
**Sunnybrook total**	**Sum of Squares**	**df**	**Mean Square**	**F**	**p**	**Sum of Squares**	**df**	**Mean Square**	**F**	**p**
Time	3395.392	2.019	1681.989	9.407	<.001*					
Time*sex	424.921	2.019	210.495	1.177	0.321	176.082	1	176.082	0.427	0.522
Time*age	87.890	2.019	43.539	0.244	0.787	55.015	1	55.015	0.133	0.719
Time between paralysis and anastomosis	109.395	2.019	54.191	0.303	0.742	393.416	1	393.416	0.954	0.342
Residuals	6135.793	34.317	178.795			7008.452	17	412.262		
**Simmetry at rest**	**Sum of Squares**	**df**	**Mean Square**	**F**	**p**	**Sum of Square**	**df**	**Mean Square**	**F**	**p**
Time	157.015	2.992	52.472	3.088	0.035*					
Time*sex	150.589	2.992	50.324	2.962	0.041*	20.070	1	20.070	0.441	0.516
Time*age	4.724	2.992	1.579	0.093	0.963	5.655 × 10^–4^	1	5.655 × 10^–4^	1.243 × 10^–5^	0.997
Time between paralysis and anastomosis	8.050	2.992	2.690	0.158	0.923	55.388	1	55.388	1.217	0.285
Residuals	864.377	50.870	16.992			773.617	17	45.507		
**Voluntary Movement**	**Sum of Square**	**df**	**Mean Square**	**F**	**p**	**Sum of Square**	**df**	**Mean Square**	**F**	**p**
Time	2345.929	2.075	1130.416	8.988	<.001*					
Time*sex	163.190	2.075	78.635	0.625	0.547	96.186	1	96.186	0.314	0.582
Time*age	145.631	2.075	70.174	0.558	0.584	35.922	1	35.922	0.117	0.736
Time between paralysis and anastomosis	94.373	2.075	45.475	0.362	0.707	166.789	1	166.789	0.545	0.470
Residuals	4437.185	35.280	125.771			5203.733	17	306.102		
**Synkinesis**	**Sum of Square**	**df**	**Mean Square**	**F**	**p**	**Sum of Square**	**df**	**Mean Square**	**F**	**p**
Time	9.656	3.147	3.069	0.780	0.516					
Time*sex	12.912	3.147	4.103	1.043	0.383	0.544	1	0.544	0.057	0.814
Time*age	11.168	3.147	3.549	0.902	0.450	4.287	1	4.287	0.448	0.512
Time between paralysis and anastomosis	9.924	3.147	3.154	0.802	0.504	0.161	1	0.161	0.017	0.898
Residuals	210.468	53.494	3.934			162.619	17	9.566		

Abbreviations:

Time*sex: Sex-corrected data

Time*age: Age-corrected data

df: Degrees of Freedom

F: F-value

p: p-value

p*: statistically significant difference (p < 0.05)

### Comparison between study group and control group

**Fig. 3:**
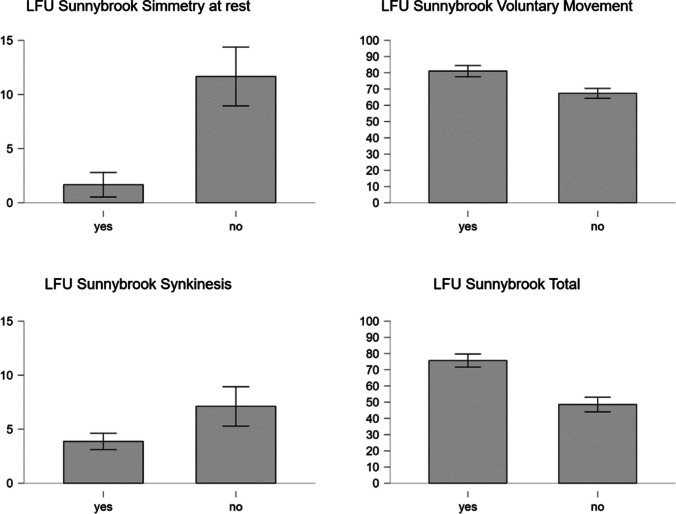
Comparison between study group and control group. **LFU**: last follow-up Sunnybrook score. **Yes**: patients rehabilitated with NMR (study group); **No**: patients not rehabilitated with NMR (control group)

The study group had a greater mean age (51.9 vs 43.2) and a longer interval between paralysis onset and HFC (238.6 vs 122.6 days), while gender distribution was similar. Nonetheless, at the last follow-up the study group showed significantly better Sunnybrook scores across all domains (p < 0.001), particularly for symmetry at rest, which resulted close to normal symmetry in the study group (1.7 vs 11.7 out of 20), and for synkinesis which were almost half of the control group (3.8 vs 7.1 out of 15) (Table 3). Box plots illustrate these comparisons (Fig. 3).

**Table 3:** Comparison between study group and control group

	**Study group**	**Control group**	
**Sunnybrook score**	**Mean (SD)**	**Mean (SD)**	**p**
Sunnybrook total	75.667 (10.845)	48.556 (5.855)	< 0,001*
Sunnybrook at rest	1.667 (3.032)	11.667 (3.536)	< 0,001*
Sunnybrook voluntary movements	81.067 (9.214)	67.333 (4.000)	< 0,001*
Sunnybrook synkinesis	3.867 (2.030)	7.111 (2.369)	< 0,001*
**House—Brackmann score**	2.93 (0.58)	3.34 (0.50)	0.038*
**FDI physic**	69.6 (17.1)	70.0 (11.5)	0.383
**FDI social**	77.2 (14.7)	74.3 (4.73)	0.144
**MDADI global**	3.0 (1.15)	2.1 (1.74)	0.836
**MDADI composite**	71.2 (12.3)	70.8 (14.9)	0.210

Abbreviations:

FDI: Facial Disability Index

MDADI: MD Anderson dysphagia inventory

SD: standard deviation

p: p-value

p*: statistically significant difference (p < 0.05)

Questionnaire completion rates differed substantially between groups: 43% in the NMR group (13/30 patients) and 100% in the control group (9/9 patients). No statistically significant differences were observed between groups for either the functional or social subscales of the Facial Disability Index (FDI) or for the global and composite scores of the MD Anderson Dysphagia Inventory (MDADI) (Table 3).

## Discussion

End-to-end HFC provides maximal axonal input and access to the extensive hypoglossal motor cortex. This concept becomes apparent when observing at the “homunculus motorius” of Penfield and Rasmussen, which illustrates the large hypoglossal area [16, 17].

The beneficial effects observed with NMR may reflect, at least in part, mechanisms related to cortical reorganization and motor relearning, rather than direct modification of peripheral nerve regeneration. These effects are consistent with the known capacity of the central nervous system to adapt following peripheral nerve transfer, although no direct neurophysiological measurements were performed in the present study [18]. While symmetry at rest improves sharply until 12 months and stabilizes into perfect symmetry, voluntary movements continue their improvement up to 18 months and then stabilize in good to very good values (Fig. 1). The expected synkinesis increase between 6 to 12 months is limited and stabilizes thereafter in all patients.

Female patients showed an improving trend for synkinesis after 12 months; however, these differences did not reach statistical significance and should be interpreted cautiously (Fig. 2). Synkinesis have been identified in almost all patients undergoing hypoglossal-facial transfer in the literature. They have been attributed to a mixing of the direction of regrowing axons, resulting in a chaotic and excessive innervation of facial muscles. Based on our results, we may hypothesize that the reorganization of the hypoglossal cortical motor area occurring after 12 to 24 months, guided by NMR, may permit a better control of the output in the single axons, eventually improving synkinesis and symmetry at rest.

Indeed, the overall score improves up to 18 months of NMR treatment and maintain stable in the 24 months evaluation. Patients of the control group showed increased muscle tone or contracture at rest, thus resulting in worst symmetry at rest. Synkinesis were significantly worst in this group, resulting also in a decreased range of voluntary movement due to the synkinetic activation of antagonist muscles.

The efficacy of NMR after HFC was demonstrated in our study through the comparison between the study with the control group (Fig. 3). We observed that either the overall value or the single domains of the Sunnybrook scale resulted significantly better in the NMR group compared with the control one. In particular, the best results were observed for the symmetry at rest and synkinesis, which are specific targets of the NMR.

The use of the Sunnybrook scale is a strength of our study, as it makes the evaluation of results and the comparison more precise, giving more insight into the different aspects of facial palsy. The main limitation of House – Brackmann scale is the impossibility to distinguish between mild, moderate or severe synkinesis, and between the lower and upper part of the face. In our study, indeed, we observed an average grade 3 after 12 months, without any variation afterwards for the study group. We should mention that, interestingly, in the study group, some patients reached a grade 2 of the HB scale, due to absent or complete reduction of synkinesis. When comparing the two groups with HB scale, even if we still found a significant difference in favour of the NMR group, it was less important than what we found with the Sunnybrook scale. In addition, most of the studies of the literature lack in reporting a precise timing of outcome measures and the type and length of physical rehabilitation after surgery is frequently not clearly reported.

Another strength of the present study are the long-term rehabilitation period and periodic evaluation of patients. This aspect is essential when assessing rehabilitation methods acting on neural plasticity, which require long time to occur.

We included the age and the time between paralysis and HFC in all the ANOVA models, as they have been reported to be factors impacting on the final outcome by several authors. Nonetheless, in our analysis we did not find any significant impact of the time between paralysis and HFC, age and gender on the final result or the evolution of the Sunnybrook scale and its domains during the 24 months rehabilitation period.

When analysing patient reported outcomes by means of the FDI scale, surprisingly, we observed no significant differences between the groups in both the functional score and the social score. We can speculate that patients undergoing an intensive rehabilitation program may be more critical regarding the facial palsy outcomes compared with the patients of the control group.

However, several limitations are present in the literature as regards the analysis of tongue tonicity and swallowing problems after HFC, because all the studies rely on a subjective evaluation or not validated scales [19, 20]. In the present study we used the MDADI scale to assess the swallowing after HFC treated with NMR and speech therapy for the tongue. To the authors knowledge, this is the only study in the literature measuring swallowing outcomes with the MDADI scale. We observed better scores both for global and composite scores in the study group compared with the control group, however the difference was not significant and this can be associated with the small groups (13 patients from the study group and 9 of the control group completed the MDADI questionnaire) and some selection bias cannot be excluded. Nonetheless, average results in the study group were good to very good, both for the global and composite scores.

This study has several important limitations. Its retrospective design limits causal inference and is subject to selection and information bias. The control group was small and highly heterogeneous, comprising patients treated with non-standardized rehabilitation protocols of unknown intensity, duration, and supervision. This heterogeneity prevents direct comparison of specific therapeutic effects and limits the ability to draw definitive conclusions regarding the superiority of NMR over other approaches.

Baseline differences between groups, including age, etiology, and time from paralysis to surgery, may have introduced residual confounding despite adjustment in the statistical models. In addition, the NMR group was followed longitudinally with repeated assessments, whereas the control group was evaluated only at a single late follow-up visit, further limiting temporal comparisons.

Patient-reported outcomes were affected by missing data, as only 43% of NMR patients returned the FDI and MDADI questionnaires, compared with full completion in the control group. This introduces a potential response bias, as patients with worse outcomes may have been less likely to respond.

Finally, subgroup analyses, including sex-based comparisons, were underpowered, and the results should be considered exploratory. These findings require confirmation in larger prospective studies with standardized rehabilitation protocols.

## Conclusion

In conclusion, we observed significantly improved outcomes in patients treated with NMR after end-to-end HFC, compared to those not treated with NMR, in the long term. The results demonstrate that there is still an unexplored potential of the end-to-end HFC surgical technique in treating facial palsy, that can be used through rehabilitation methods acting on cortical plasticity. Larger studies including the HFC surgical variants, may permit to confirm our results and to compare results of NMR among technical variants.

## Data Availability

The datasets generated during and/or analysed during the current study are available from the corresponding author on reasonable request.
